# Transcriptome and Metabolome Analyses Provide Insights into the Stomium Degeneration Mechanism in Lily

**DOI:** 10.3390/ijms222212124

**Published:** 2021-11-09

**Authors:** Ling He, Xinyue Liu, Ze Wu, Nianjun Teng

**Affiliations:** 1 Key Laboratory of Landscaping Agriculture, Ministry of Agriculture and Rural Affairs, College of Horticulture, Nanjing Agricultural University, Nanjing 210095, China; 2018204044@stu.njau.edu.cn (L.H.); 2019104105@njau.edu.cn (X.L.); wuze@njau.edu.cn (Z.W.); 2 Key Laboratory of Biology of Ornamental Plants in East China, National Forestry and Grassland Administration, College of Horticulture, Nanjing Agricultural University, Nanjing 210095, China; 3 Jiangsu Graduate Workstation of Nanjing Agricultural University and Nanjing Oriole Island Modern Agricultural Development Co., Ltd., Nanjing 210043, China

**Keywords:** *Lilium* spp., anther dehiscence, stomium degeneration, transcriptome, metabolome

## Abstract

Lily (*Lilium* spp.) is a widely cultivated horticultural crop that has high ornamental and commercial value but also the serious problem of pollen pollution. However, mechanisms of anther dehiscence in lily remain largely unknown. In this study, the morphological characteristics of the stomium zone (SZ) from different developmental stages of ‘Siberia’ lily anthers were investigated. In addition, transcriptomic and metabolomic data were analyzed to identify the differentially expressed genes (DEGs) and secondary metabolites involved in stomium degeneration. According to morphological observations, SZ lysis occurred when flower buds were 6–8 cm in length and was completed in 9 cm. Transcriptomic analysis identified the genes involved in SZ degeneration, including those associated with hormone signal transduction, cell structure, reactive oxygen species (ROS), and transcription factors. A weighted co-expression network showed strong correlations between transcription factors. In addition, TUNEL (TdT-mediated dUTP nick-end labeling) assays showed that programmed cell death was important during anther SZ degeneration. Jasmonates might also have key roles in anther dehiscence by affecting the expression of the genes involved in pectin lysis, water transport, and cysteine protease. Collectively, the results of this study improve our understanding of anther dehiscence in lily and provide a data platform from which the molecular mechanisms of SZ degeneration can be revealed.

## 1. Introduction

Lily (*Lilium* spp.) is highly valued for its attractive variations in fragrance, pattern, and color, and holds a high market share in the global cut flower industry [1,2]. Control of male fertility is essential for plant reproduction and selective breeding [3]. On the other hand, pollen can also have serious negative effects. For example, many ornamental plants produce excessive amounts of pollen, including *Populus*, lily, and chrysanthemum, and exposure to high amounts of pollen can lead to an anaphylactic reaction in susceptible populations [4,5,6]. Moreover, large amounts of pollen are not necessary for the commercial value of ornamental plants. Therefore, sterile male or pollen-free ornamental plants can be very valuable. Although lily is an important commercial flower crop, its market popularity and landscape application are adversely affected by severe pollen pollution. Many studies have focused on anther development in model plants, but there has been little research on flower crops, and although pollen development in lily has been examined in recent years [5,7], the mechanisms of anther dehiscence remain largely unknown.

In plant evolution, selfing leads to genetic decline in offspring. Differences in stamen fertility can be an important factor in heredity and evolution. In *Arabidopsis*, late anther sterility involves anther dehiscence, pollen maturation, and filament elongation [8]. Anther dehiscence and pollen maturation occur after the anther middle layer is crushed and meiosis is completed [9]. Anther dehiscence is a multistage process that involves differentiation of specialized cells and changes in water status [3].

Endothecium cell secondary thickening is also critical in anther dehiscence [10]. Regulation of the thickening is controlled by several transcription factors, such as MYB26, MYB46, MYB108, NST1/NST2, and NSD1 [10,11,12,13,14].

Plant hormones also affect dehiscence. The *Arabidopsis arf17* mutant has defective endothecium thickening and male sterility, and ARF17 can directly regulate *MYB108*, which also has functions in anther endothecium thickening [15]. Similarly, anthers of *Arabidopsis tir1 afb2 afb3* triple and *tir1 afb1 afb2 afb3* quadruple mutants show early endothecium thickening [16]. At stage 10 of anther development, lignification, which leads to anther cracking in advance, occurs in the endothecium of *afb1 opr3* anthers but not in the endothecium of wild-type plants [8]. Studies using dehiscent mutants show that JAs (JAs) also contribute to control anther dehiscence [3,17,18]. Jasmonate biosynthetic enzyme mutants, including the fatty acid desaturation (*fad*) mutant, *opr3* (mutation in 12-oxophytodienoic acid reductase) mutant, delayed-dehiscence 1 (*dde1*) and *dde2* mutant, defective in anther dehiscence 1 (*dad1*) mutant, and allene oxide synthase mutant [3], indicate JA involvement in anther indehiscence. The *lox3 lox4* double mutant has abnormal anther maturation and defective dehiscence [17]. In addition, delayed dehiscence has also been observed in mutants defective in JA perception. The JA receptor COI 1 (coronatine insensitive 1) is involved in anther fertility [18]. As a JA signal transporter, MYB21/24 interacts with both MYC and JAZ proteins, which can repress the bHLH–MYB complex activation function. However, JA induces JAZ degradation and releases the bHLH–MYB complex to subsequently activate expression of downstream genes for anther dehiscence development [19,20].

Mechanisms regulating anther dehiscence have been studied in model plants, but the focus has largely been limited to endothecium cell lignification [3,10,11,12,13,14,15]. Few studies have examined stomium zone (SZ) degeneration. Molecular regulation mechanisms of anther SZ degeneration remain largely unknown. In this study, SZ degeneration was investigated in the large anthers of lily using a combination of morphological, physiological, and comparative transcriptomic and metabolomic analyses. The aim of the research was to understand SZ degeneration at different stages of anther development at the morphological, physiological, and molecular levels. Transcriptomic analysis of the five developmental stages of SZ tissues was conducted to identify the differentially expressed genes (DEGs) associated with SZ lysis, and metabolomic analysis was conducted to explore differences in metabolism during SZ degeneration. The results will provide new insights into the regulation of SZ lysis and help to screen new factors to control anther dehiscence.

## 2. Results

### 2.1. Occurrence of Anther Dehiscence in Lily Flower Buds 6–8 cm in Length

**Figure 1:**
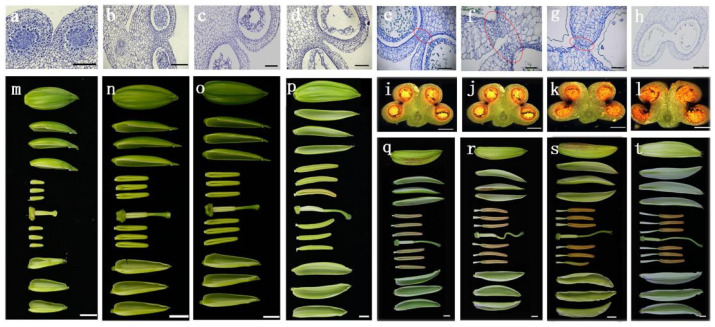
Changes in flower structure as flowers buds develop from 2 to 9 cm in length. (**a**–**h**) Histochemical analysis of anther stomium zone structure. (**a**–**d**) Anthers in flower buds from 2 to 5 cm in length. No stomium zone cell degeneration occurs. Bar = 200 μm. (**e**–**h**) Stomium lysis occurs in flower buds from 6 to 9 cm in length. Bar = 200 μm. (**i**–**l**) Anther cross section of e–h. Anther dehiscence occurs in flower buds after 8 cm in length. Bar = 200 μm. (**m**–**t**) Flower phenotype during developmental stages in a–h. Bar = 1000 mm. Red elliptical line in (**e**–**g**) represents the sampling position of the stomium zone.

The Oriental hybrid lily ‘Siberia’ was used to investigate the relation between SZ degradation and flower development. Developmental periods of lily anthers were separated according to flower bud length. Stages of flower buds at 2–9 cm in length were designated as S2–S9 cm. In stages S2–S5 cm, SZ degeneration was not observed in anthers (Figure 1a–d). SZ lysis occurred during stages S6–S8 cm (Figure 1e–g) and was completed at stage S9 cm (Figure 1h). In addition, to further observe the morphology of developing flower buds, stamens, pistils, and perianths, the morphology of the whole flower during stages S6–S9 cm is shown in Figure 1i–t. Pigment accumulation in the SZ was observed in stages S6–S9 cm (Figure 1i–l). These results indicated that stages S6–S8 cm were the critical ones in SZ lysis.

### 2.2. Regulation of Stomium Zone Degradation Showing Fewer Differentially Expressed Genes

The SZ samples were collected by cutting anthers freehand, which were then immediately frozen in liquid nitrogen and stored at −80 °C for transcriptome sequencing. Based on FPKM (fragments per kb per million reads), the gene expression levels were explored in the anther SZ during five developmental stages (S5–S9 cm) using RNA-seq. More than 755.56 million clean reads were generated from 15 samples. The Q30 rates of the 15 libraries ranged from 89.24% to 94.90%. The GC rates of the 15 libraries ranged from 49.02% to 50.22%. Results are shown in Table S1.

**Figure 2:**
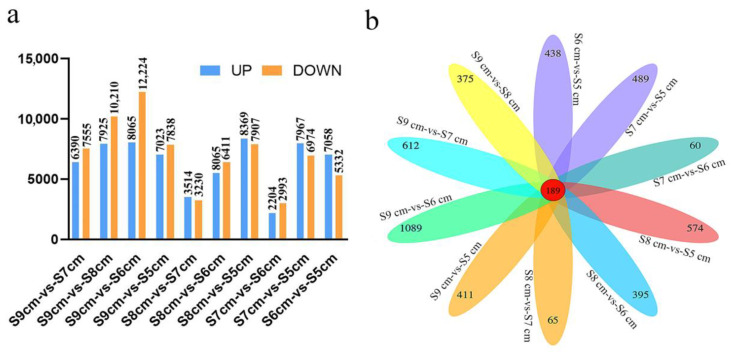
Summary of genes in the anther stomium zone during different developmental stages of lily flower buds. (**a**) Numbers of upregulated and downregulated differentially expressed genes (DEGs) in pairwise comparisons of five developmental stages. (**b**) Venn diagram of the number of DEGs in 10 pairwise comparisons: S9 cm-vs-S8 cm, S9 cm-vs-S7 cm, S9 cm-vs-S6 cm, S9 cm-vs-S5 cm, S8 cm-vs-S7 cm, S8 cm-vs-S6 cm, S8 cm-vs-S5 cm, S7 cm-vs-S6 cm, S7 cm-vs-S5 cm, and S6 cm-vs-S5 cm.

Differentially expressed genes in the anther SZ from the five developmental stages were identified. DEGs were identified after comparisons of the FPKM values for each gene between different SZ developmental periods. Numbers of DEGs were as follow: 6390 up- and 7555 downregulated DEGs in S9 cm-vs-S8 cm; 7925 up- and 10,210 downregulated DEGs in S9 cm-vs-S7 cm; 8065 up- and 12,224 downregulated DEGs in S9 cm-vs-S6 cm; 7023 up- and 7838 downregulated DEGs in S9 cm-vs-S5 cm; 3514 up- and 3230 downregulated DEGs in S8 cm-vs-S7 cm; 5513 up- and 6411 downregulated DEGs in S8 cm-vs-S6 cm; 8369 up- and 7907 downregulated DEGs in S8 cm-vs-S5 cm; 2204 up- and 2993 downregulated DEGs in S7 cm-vs-S6 cm; 7967 up- and 6974 downregulated DEGs in S7 cm-vs-S5 cm; and 7058 up- and 5332 downregulated DEGs in S6 cm-vs-S5 cm (Figure 2a). These results indicated that there were relatively small differences in DEGs during stages S6–S8 cm.

Overlap of DEGs is shown in a Venn diagram (Figure 2b). Among the DEGs, only 189 were common to the five stages, which indicated that there were different response mechanisms involved in the regulation of anther dehiscence during SZ degradation.

### 2.3. Classification Categories of Differentially Expressed Genes Involved in Anther Dehiscence

All unigenes (46,794) were annotated using seven public functional databases, including 26,962 (57.62%) with Nr; 20,693 (44.22%) with Swissprot; 6044 (12.92%) with KEGG; 15,862 (33.90%) with KOG; 21,587 (52.67%) with eggNOG; 18,021 (38.51%) with GO; and 18,445 (39.42%) with Pfam (Table S2).

The largest and smallest numbers of annotated unigenes were obtained from the Nr and KEGG databases, respectively. With Nr annotation, the distribution of annotated species was determined, and 24.69%, 19.41%, 5.73%, 4.98%, 4.67%, 2.50%, 2.04%, 1.85%, 1.76%, and 1.38% unigenes were closely matched with *Elaeis guineensis*, *Phoenix dactylifera*, *Asparagus officinalis*, *Ananas comosus*, *Musa acuminata*, *Musa balbisiana*, *Ensete ventricosum*, *Arabidopsis thaliana*, *Dendrobium catenatum*, and *Vitis vinifera*, respectively. The proportion of other species was 23.28% (Figure S1).

**Figure 3:**
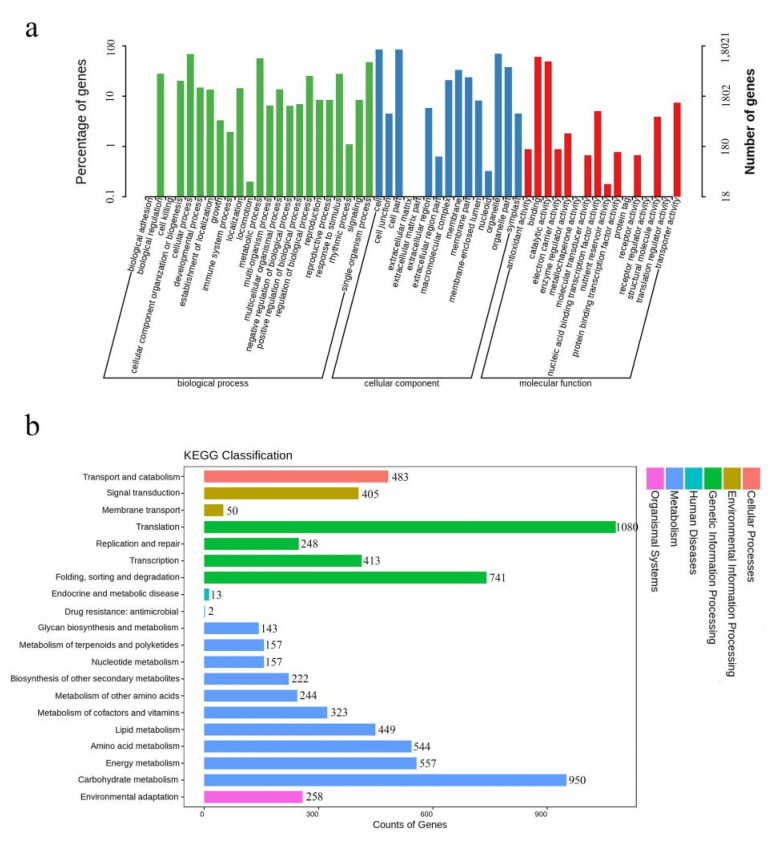
GO and KEGG classifications of differentially expressed genes. (**a**) GO (Gene Ontology) classification of differentially expressed genes (DEGs). The *x*-axis represents GO terms, and the *y*-axis represents the number of DEGs. (**b**) KEGG (Kyoto Encyclopedia of Genes and Genomes) classification of DEGs. The *x*-axis represents the number of DEGs, and the *y*-axis represents the KEGG terms.

To further explore the function of the 18,012 assembled genes, GO (Figure 3a) term enrichment analysis was performed, which has three categories: biological process, cellular component, and molecular function. In the biological process category, “cellular process” was the most highly represented term, followed by “metabolic process” and “single-organism process”. In the cellular component category, the main functional terms were “cell”, “cell part”, and “organelle”. In the molecular function category, the most enriched terms were “binding”, “catalytic activity”, and “transporter activity” (Figure 3a).

The four largest representative classification categories of KEGG (Figure 3b) were “translation (1080, 17.87%)”, “carbohydrate metabolism (950, 15.72%)”, “folding, sorting and degradation (742, 12.26%)”, and “energy metabolism (557, 9.21%)”. A few unigenes were involved in “drug resistance: antimicrobial”, “endocrine and metabolic disease”, and “membrane transport.” Additionally, “starch and sucrose metabolism (176, 2.91%; pathway: ko00500)”, “ascorbate and adorate metabolism (57, 0.94%; pathway: ko00053)”, “alpha-linolenic acid metabolism (56, 0.93%; pathway: ko00592)”, “flavonoid biosynthesis (41, 0.68%; pathway: ko00941)”, “phenylalanine metabolism (33, 0.55%; pathway: ko00360)”, “zeatin biosynthesis (18, 0.30%; pathway: ko00908)”, “brassinosteroid biosynthesis (13, 0.22%; pathway: ko00905)”, “flavone and flavanol biosynthesis (9, 0.15%; pathway: ko00944)”, and “anthocyanin biosynthesis (3, 0.05%; pathway: ko00942)”, might also be associated with anther dehiscence.

### 2.4. Jasmonate Biosynthesis and Signal Transduction Roles in Stomium Zone Degradation

**Figure 4:**
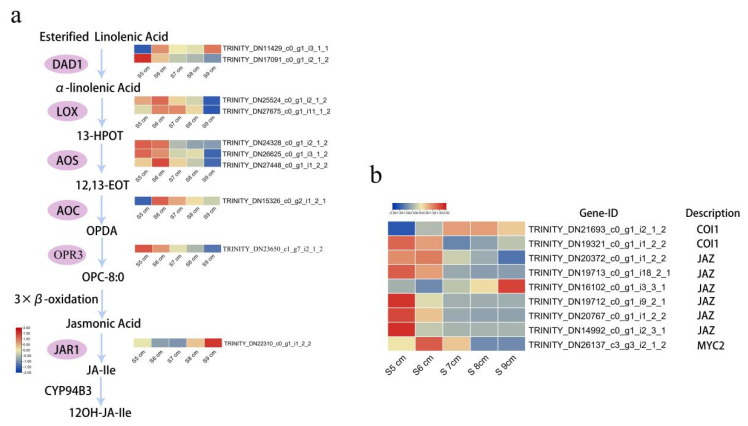
Differentially expressed genes associated with jasmonate biosynthesis and signal transduction. (**a**) Heat maps of the expression patterns of the differentially expressed genes (DEGs) associated with JA biosynthesis. (**b**) Heat maps of the DEGs involved in JA signal transduction. Red rectangles represent upregulated genes, and blue rectangles represent downregulated genes. All genes in this diagram are listed in Table S3.

To identify key genes involved in SZ degeneration in lily anthers, expression patterns of phytohormone-related DEGs (S9 cm-vs-S5 cm) were analyzed based on their function. The two largest classifications were auxin and JA signal transduction (Table S3). Auxin signal transduction includes many members, such as auxin influx carrier protein AUX1, auxin-responsive protein IAA, auxin responsive GH3 family proteins, and SAUR family proteins. During the five developmental stages, *AUX1* (TRINITY_DN25809_c0_g1_i6_1_2) and *transport inhibitor response 1* (TRINITY_DN23585_c0_g1_i1_2_2) had highest levels of expression at stage S6 cm. Among four auxin-responsive protein IAA genes, three genes (TRINITY_DN18566_c0_g3_i2_1_2, TRINITY_DN7371_c0_g1_i1_1_1, and TRINITY_DN6422_c0_g1_i1_1_1) had highest expression levels at stage S5 cm and one (TRINITY_DN21346_c0_g1_i3_2_2) at stage S9 cm. Furthermore, four *SAURs* (TRINITY_DN3438_c0_g1_i1_1_2, TRINITY_DN16038_c0_g1_i2_2_2, TRINITY_DN21146_c0_g1_i2_2_2, and TRINITY_DN16905_c0_g1_i1_3_1) had higher FPKM values at stages S5 cm, S7 cm, and S9 cm. In Figure 4, ten DEGs associated with JAs synthetase, *JAZs* (jasmonate ZIM domain-containing protein genes), and *MYC2* were classified in JAs signal transduction. *MYC2* (TRINITY_DN26137_c3_g3_i2_1_2) was most highly expressed at stage S6 cm. Four *JAZs* (TRINITY_DN19713_c0_g1_i18_2_1, TRINITY_DN19712_c0_g1_i9_2_1, TRINITY_DN20767_c0_g1_i1_2_2, and TRINITY_DN14992_c0_g1_i2_3_1) were highly expressed at stage S5 cm. Two *JAZs*, TRINITY_DN20372_c0_g1_i1_2_2 and TRINITY_DN16102_c0_g1_i3_3_1, were highly expressed at stages S6 cm and S9 cm, respectively. Jasmonate synthetase gene *JAR1* (TRINITY_DN22310_c0_g1_i1_2_2) was most highly expressed in stage S9 cm. *DAD1* (TRINITY_DN17091_c0_g1_i2_1_2), *LOXs* (TRINITY_DN25524_c0_g1_i2_1_2 and TRINITY_DN27675_c0_g1_i11_1_2), *AOSs* (TRINITY_DN24328_c0_g1_i2_1_2, TRINITY_DN26625_c0_g1_i3_1_2, TRINITY_DN27448_c0_g1_i1_2_2), and *AOC* (TRINITY_DN15326_c0_g2_i1_2_1) and OPR3 (TRITY_DN22310_c0_g1_i1_2_2) were most highly expressed during stages S5 cm and S6 cm. The results indicated that JA biosynthesis and signal transduction play roles before SZ degeneration.

### 2.5. Genes Associated with Cysteine Proteinases during Stomium Zone Degradation

**Figure 5:**
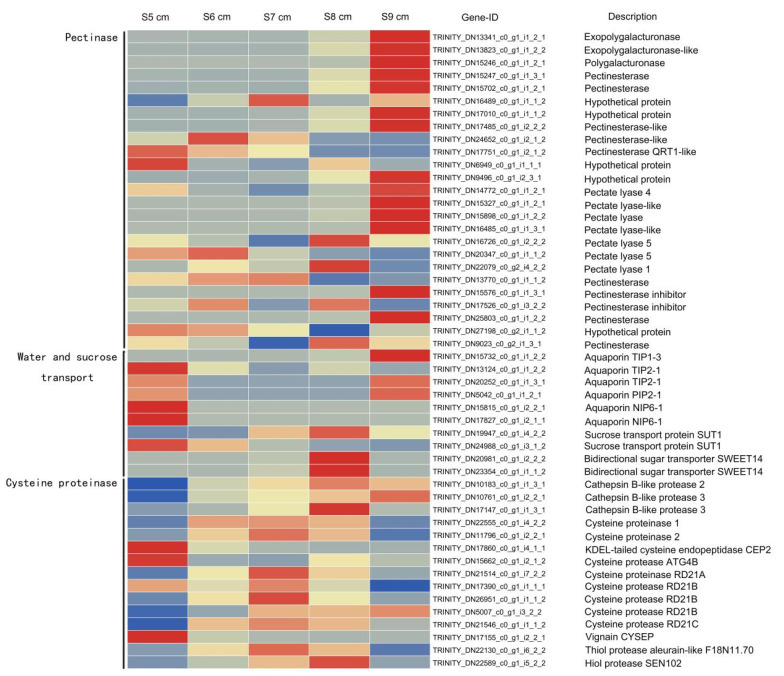
Heat maps of the differentially expressed genes associated with pectinase, water and sucrose transport, and cysteine proteinases.

Genes associated with pectin degeneration, water and sucrose transport, and programmed cell death (PCD) were differentially expressed during SZ degradation (Table S3). Pectin metabolic genes involved in cell wall degeneration were identified, including *exopolygalacturonase*, *polygalacturonase*, *pectinesterase*, *hypotheticalprotein*, *pectinesterase-like*, *pectate lyase*, *pectate lyase-like*, and *pectinesterase inhibitor*. The 25 genes associated with pectin degeneration were mostly expressed in stage S9 cm (Figure 5). Water and sucrose transport genes included those of several aquaporins and sucrose transport proteins. Six *aquaporins* were primarily expressed in stage S5 cm, but sucrose transport protein genes were highly expressed in stage S8 cm. Five PCD-related genes were identified, including *athepsin B-like protease*, *cysteine proteinase*, and those of other proteases. High expression levels of PCD-related genes were primarily in stages S6 cm and S7 cm. This result suggested that the 15 protease genes significantly affected SZ cell death.

### 2.6. Transcription Factors Roles in Anther Dehiscence

**Figure 6:**
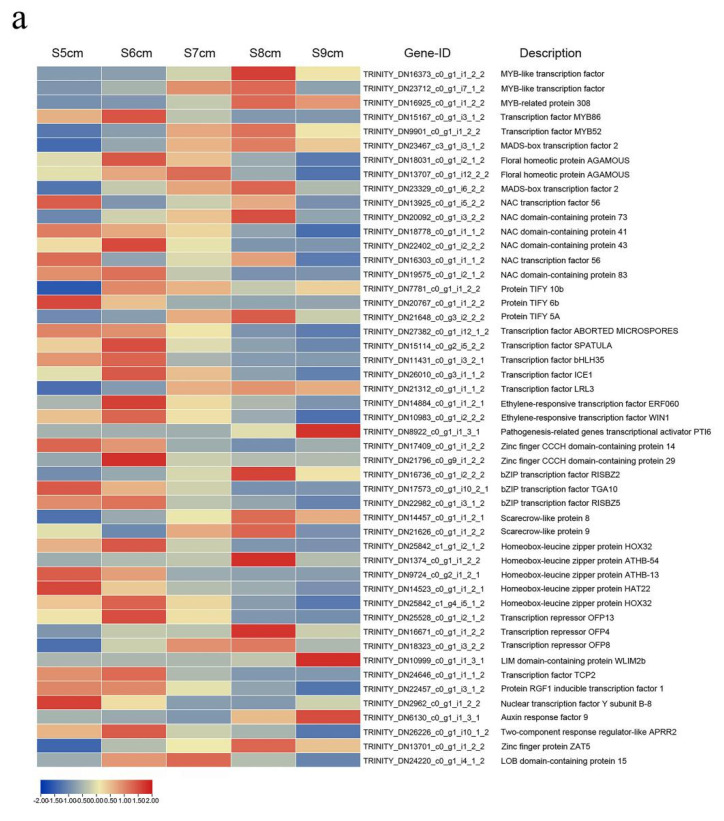

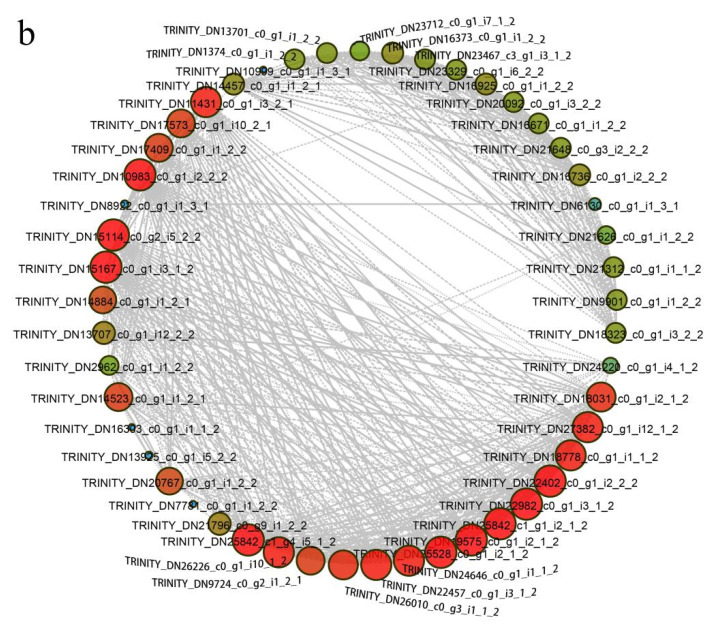
Heat maps and co-expression network of the transcription factors (TOP49). (**a**) Heat maps of the transcription factors (TFs). Red rectangles represent upregulated genes, and blue rectangles represent downregulated genes. (**b**) Co-expression network of TFs. Nodes represent TFs. A solid line edge indicates higher correlation, whereas a dotted line edge indicates lower correlation. The size and color of the nodes are determined by the degree of connectivity. The correlation determines the width of the edges.

Many studies found that anther dehiscence is governed by special transcription factors (TFs), which are the master regulators controlling how anther development-related genes are turned off and on [10,13,15]. The 49 differentially expressed TFs in the group S8 cm-vs-S5 cm were examined (Figure 6a and Table S4). In the MYB TF family, four TFs were highly expressed in stage S8 cm, and one TF (TRINITY_DN15167_c0_g1_i3_1_2) was highly expressed in stage S6 cm. In the MADS TF family, TFs were highly expressed from stages S6 cm to S9 cm. In the NAC TF family, three TFs were highly expressed in stage S5 cm, two TFs were highly expressed in stage S6 cm, and one TF was highly expressed in stage S8 cm. In the TIFY TF family, three TFs were most highly expressed in stages S5 cm, S6 cm, and S8 cm. Among five bHLH TFs, three TFs were most upregulated in stage S6 cm, whereas the other two TFs had maximal FPKM values in stages S5 cm and S8 cm. Two AP2/ERF TFs had maximal FPKM values in stage S6 cm. Three C3H family members had maximal FPKM values in stages S5 cm, S6 cm, and S9 cm. Three bZIP family TFs had maximal FPKM values in stages S5 cm, S6 cm, and S8 cm. Two GRAS family TFs were highly expressed in S8 cm. Eight TFs from the HB-HD-ZIP and OFP families had significantly higher expression in stages S5 cm, S6 cm, and S8 cm. Eight other TFs in LIM, TCP, PLATZ, NF-YB, B3-ARF, GARP, C2H2, and LOB families were also identified. The highest FPKM values of those TFs were in stages S5 cm to S9 cm. They were initially upregulated during anther dehiscence. The results suggested TF genes regulated SZ degeneration.

Based on the expression levels, correlations between the 49 TFs were analyzed (Figure S2). Among the TFs, there were more significant positive correlations than negative correlations. A weighted network of the TFs was constructed on the basis of their expression patterns. Forty-nine nodes and 1176 co-expressed gene pairs were identified. Because the gene pairs with a *p*-value < 0.05 were considered to show consistent correlations, they were then used in the weighted network construction (Figure 6b). As shown in Figure 6, there were many interconnections among the TFs, which indicated that SZ degeneration was a coordinated process. Among the TFs, ethylene-responsive transcription factor WIN1 (TRINITY_DN10983_c0_g1_i2_2_2), transcription factor SPATULA (TRINITY_DN15114_c0_g2_i5_2_2), transcription factor MYB86 (TRINITY_DN15167_c0_g1_i3_1_2), homeobox-leucine zipper protein HOX32 (TRINITY_DN25842_c1_g1_i2_1_2), transcription repressor OFP13 (TRINITY_DN25528_c0_g1_i2_1_2), NAC domain-containing protein 83 (TRINITY_DN19575_c0_g1_i2_1_2), NAC domain-containing protein 43 (TRINITY_DN22402_c0_g1_i2_2_2), and bZIP transcription factor RISBZ5 (TRINITY_DN22982_c0_g1_i3_1_2) had higher connectivity than that of the other TFs.

In addition, low connectivity was found for LIM domain-containing protein WLIM2b (TRINITY_DN10999_c0_g1_i1_3_1), auxin response factor 9 (TRINITY_DN6130_c0_g1_i1_3_1), protein TIFY 10 b (TRINITY_DN7781_c0_g1_i1_2_2), NAC transcription factor 56 (TRINITY_DN13925_c0_g1_i5_2_2), NAC transcription factor 56 (TRINITY_DN16303_c0_g1_i1_1_2), and pathogenesis-related genes transcriptional activator PTI6 (TRINITY_DN8922_c0_g1_i1_3_1), as indicated by lower correlations with other TFs. Thus, interconnections among TFs should be considered when investigating the regulation of SZ degeneration.

### 2.7. Flavone and Flavanol Biosynthesis Is Enriched in the Critical Degeneration Stage of the Stomium Zone

**Figure 7:**
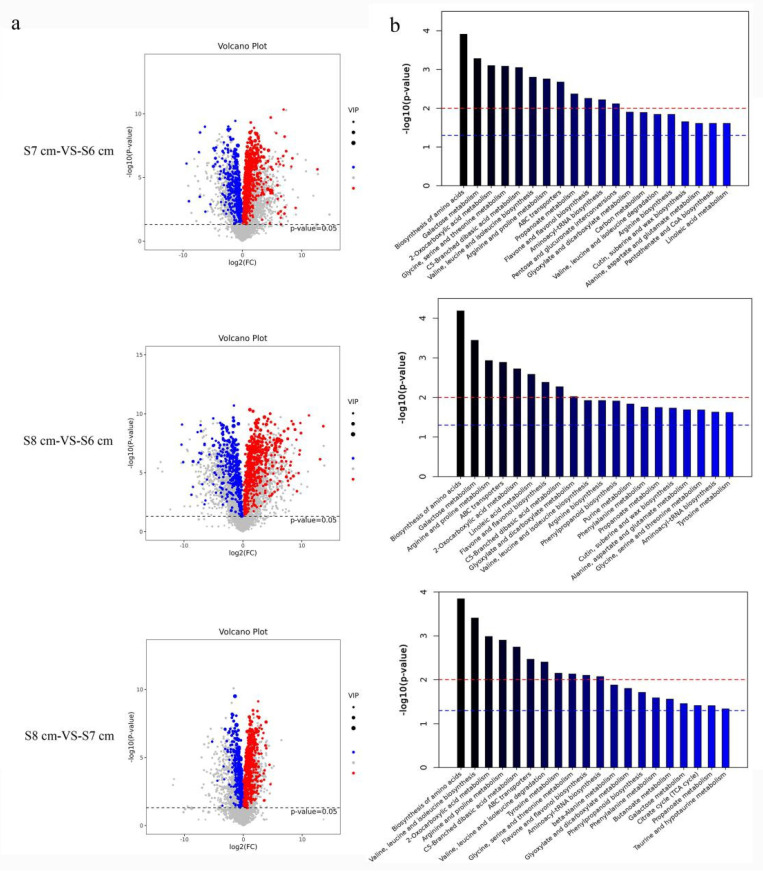
Volcano map of the differentially expressed metabolites (DEMs) and KEGG pathway annotations of the metabolites. (**a**) Abscissa and ordinate represent the difference in fold change and degree of significance in the difference of expression, respectively. Red dots represent upregulated DEMs, green dots represent downregulated DEMs, and gray dots represent no significant difference DEMs. (**b**) In KEGG pathway annotations of the metabolites, abscissa, and ordinate represent the KEGG pathway annotation and difference in fold change, respectively.

Stages S6 cm to S8 cm were identified as the critical ones in SZ degeneration. In the metabolomic analysis of the SZ samples (S6 cm, S7 cm, and S8 cm), 4962 metabolites were identified. To identify the differentially expressed metabolites (DEMs), four biological replicates of each development stage were analyzed. In the three comparison groups (S6 cm, S7 cm, and S8 cm), the number of metabolites that were upregulated was higher than the number of metabolites that were downregulated (Figure S2). A volcano map shows the distribution of DEMs (Figure 7a). KEGG pathway-enrichment analysis of DEMs was performed (Figure 7b) using the metabolomic data set. Comparisons of DEMs among the three developmental stages indicated that within the KEGG categories (top 20), terms related to “flavone and flavanol biosynthesis” were enriched in all three comparison groups.

### 2.8. Reactive Oxygen Species Mediates Stomium Zone Degeneration

**Figure 8:**
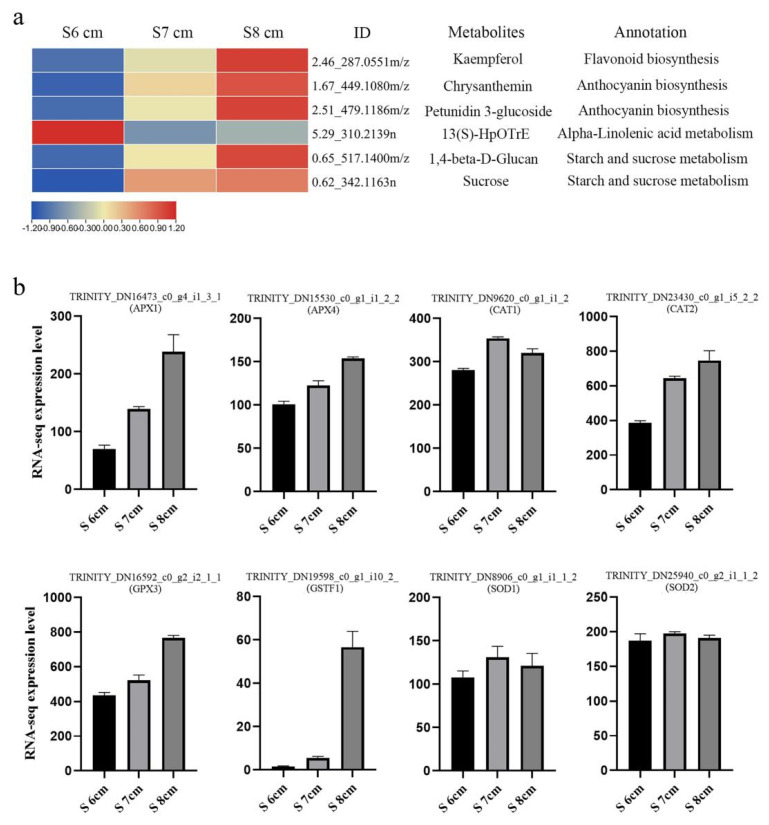
Major differentially expressed metabolites (DEMs) associated with reactive oxygen species (ROS). (**a**) Heat maps of the DEMs associated with ROS stress, including flavonoids, anthocyanins, and JAs (JA), and also the cell osmotic level. (**b**) FPKM values used in the expression analysis of the ROS marker genes in the transcriptome database. RNA-seq data are the mean of three biological replicates.

To identify the main metabolites related to SZ degeneration, a metabolites database of the enrichment pathway from S6 cm-vs-S8 cm was studied (Table S5). In Figure 8, there were four primary annotations shown. In “flavonoid biosynthesis pathways”, one metabolite (kaempferol) was highly expressed in stage S8 cm. The “anthocyanin biosynthesis pathway” was also enriched. Chrysanthemin and petunidin 3-glucoside were upregulated from stage S6 cm to S8 cm. Regarding phytohormones, one metabolite of the “alpha-linolenic acid metabolism pathway” was downregulated from stage S6 cm to S8 cm. By contrast, metabolites in the “starch and sucrose metabolism pathway” were upregulated from stage S6 cm to S8 cm. In addition, according to the FPKM values, the ROS-scavenging genes APX1, APX4, CAT2, GPX3, and GSTF1 were upregulated during the critical stage of SZ degeneration. By contrast, CAT1, SOD1, and SOD2 were upregulated and then downregulated in stages S6 cm to S8 cm. These results suggested ROS participated in SZ cell death.

### 2.9. Stomium Zone Degeneration as a Process of Programmed Cell Death

**Figure 9:**
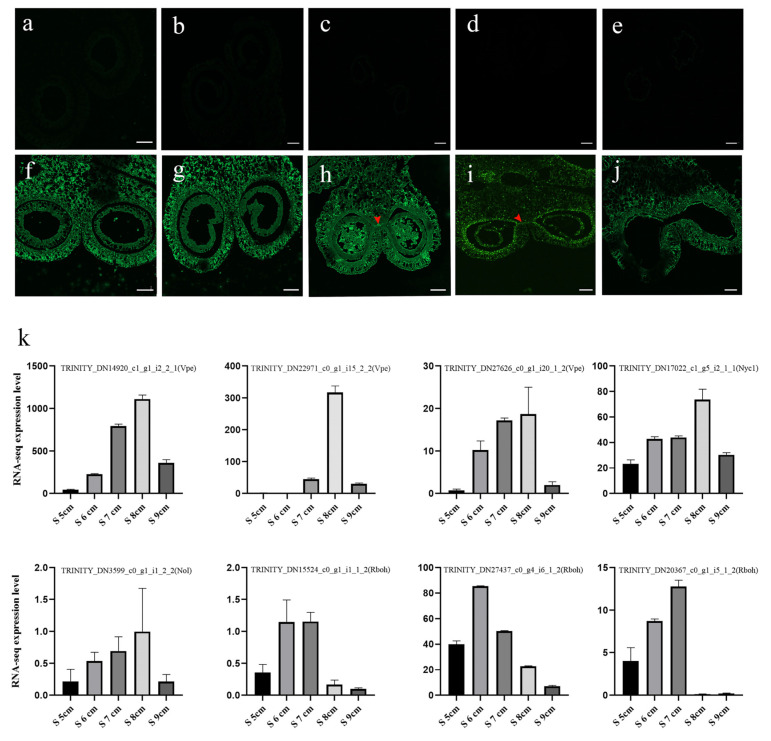
TUNEL analysis of stomium zone (SZ) degeneration in different anther developmental stages (S5 cm to S9 cm). (**a**–**e**) Control treatment of anthers at stages (**a**) S5 cm, (**b**) S6 cm, (**c**) S7 cm, (**d**) S8 cm, and (**e**) S9 cm. (**f**–**j**) Anther fluorescence at stages (**f**) S5 cm, (**g**) S6 cm, (**h**) S7 cm, (**i**) S8 cm, and (**j**) S9 cm. Green fluorescence marked by red arrows indicates a TUNEL (TdT-mediated dUTP nick-end labeling)-positive signal. The green fluorescent stain was fluorescein isothiocyanate. Bars = 200 μm. (**k**) FPKM values of the expression of the programmed cell death (PCD) marker genes in the transcriptome database. RNA-seq data are the mean of three biological replicates.

TUNEL assays were used to detect the PCD of SZ cells at different stages of anther development (S5 cm to S9 cm) (Figure 9). In the control group, a TUNEL-positive signal was not observed in the anther. In addition, a TUNEL-positive signal was not observed in the SZ at stages S5 cm and S6 cm. However, at the later S6 cm stage, positive signals were observed in SZ cells, suggesting that PCD occurred. In the S7 cm stage, additional positive signals were observed in SZ cells. At the S8 cm stage, positive signals in stomium tissues were not as strong. In the last S9 cm stage, a positive signal was barely detected in anther stomium tissues. In addition, the FPKM values of eight PCD-related genes were analyzed. In the results, vacuolar processing enzyme (Vpe) was upregulated during the critical stage of SZ degeneration, higher than the S5 cm and S9 cm expression level. Chlorophyll b reductase Nyc1 and Nol had a similar expression trend with Vpes. Respiratory burst oxidase homologue (Rboh) proteins also showed a high expression level during the critical stage of SZ degeneration. These results suggested PCD participated in SZ degeneration.

### 2.10. Jasmonates and Regulation of the Expression of Genes Associated with Stomium Zone Degeneration

**Figure 10:**
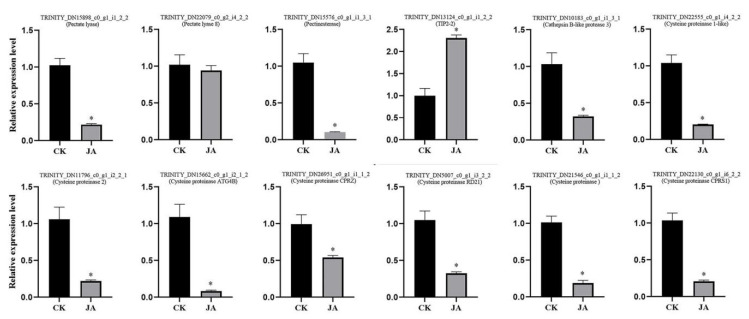
Reverse-transcription quantitative PCR of the genes associated with programmed cell death (PCD) after jasmonate treatment. Data are the mean of three replicates. CK (blank control) and JA (jasmonate) represent the 0 or 50 μM MeJA (Methyl jasmonate) treatment, respectively. “*” represent significant difference between 0 and 50 μM MeJA treatment.

Expression of genes correlated with PCD in lily was detected. In anthers with JA treatment, three genes involved in pectin degradation (TRINITY_DN15898_c0_g1_i1_2_2, TRINITY_DN22079_c0_g2_i4_2_2, and TRINITY_DN15576_c0_g1_i1_3_1) were downregulated, and one gene associated with water and sucrose transportation (TRINITY_DN13124_c0_g1_i1_2_2) was upregulated. In addition, eight genes associated with cysteine protease (TRINITY_DN15815_c0_g1_i2_2_1, TRINITY_DN22555_c0_g1_i4_2_2, TRINITY_DN11796_c0_g1_i2_2_1, TRINITY_DN15662_c0_g1_i2_1_2, TRINITY_DN26951_c0_g1_i1_1_2, TRINITY_DN5007_c0_g1_i3_2_2, TRINITY_DN21546_c0_g1_i1_1_2, and TRINITY_DN22130_c0_g1_i6_2_2) were downregulated with JAs treatment (Figure 10). Thus, JAs could control anther dehiscence by regulating cell death and water transportation.

## 3. Discussion

Studies have revealed mechanisms of anther development in different plant species [9,20,21]; however, most research has focused on anther tapetal degeneration and endothecium cell lignification. Investigations of the molecular mechanisms of anther SZ degeneration are scarce. In this study, physiological, transcriptomic, and metabolic analyses were performed in five different stages of SZ development (S5 cm, S6 cm, S7 cm, S8 cm, and S9 cm). Based on the comparative transcriptome and metabolome data (DEGs and DEMs, respectively) obtained from the different SZ developmental stages, insights into the potential molecular mechanisms underlying anther dehiscence caused by SZ degeneration are provided.

Anther opening involves forces on anther walls that facilitate rupture of the stomium [3]. Anther stomium cells undergo a PCD-related process to facilitate pollen release [22,23]. In this study, histological and morphological analyses of lily anthers showed that anther dehiscence was closely correlated with SZ degeneration (Figure 1), consistent with previous studies [9,21].

Differentially expressed genes were screened on the basis of significant differences in expression in the anther SZ at different developmental stages (Figure 2). Overall, the number of DEGs in the group related to S5 cm and S9 cm was higher than that in different groups from S6 cm to S8 cm. These results suggest the number of DEGs in S7 cm-vs-S6 cm and S8 cm-vs-S7 cm comparisons was very small. Developmental stage or environmental change can affect the number of DEGs [24]. In addition, from stage S6 cm to S8 cm in lily anther development, four microspores are released from the callose, tapetum cells gradually degenerate, and the pollen matures [21]. Consequently, stages S6 cm–S8 cm should be considered as the key period for SZ degeneration, ultimately causing anther dehiscence in lily (Figure 1).

RNA-seq is a revolutionary tool for transcriptomics, because the high-throughput sequencing technology provides an efficient and reliable platform for molecular biology research [25]. In this study, Illumina RNA-seq technology was used to study the molecular mechanisms of anther dehiscence in lily. GO term enrichment analysis was performed (Figure 3a). In the three categories of biological process, cellular component, and molecular function, the most enriched terms were “cellular process”, “cell”, and “binding”, respectively. In addition, the four largest KEGG classification categories were “translation”, “carbohydrate metabolism”, “folding, sorting and degradation”, and “energy metabolism” (Figure 3b). These results are not consistent with those in the analysis of chrysanthemum anther dehiscence. In chrysanthemums, most GO-annotated unigenes were in the categories “metabolic process”, “binding”, and “catalytic activity” [26,27]. Results vary greatly because they might be affected depending on the species and also the tissue sampled. However, the results were also consistent with the biological processes involved in anther dehiscence, such as cell dehydration, hormone biosynthesis, and TF binding.

Metabolites are intermediate and final products that have important roles in regulating plant growth and development. The total number of plant metabolites is as high as 200,000, which indicates the diversity of plant natural substances [28]. Because of the high diversity, plant metabolites are ideal targets to study the regulation of biosynthesis [29]. Genetic mechanisms underlying changes in plant metabolites have been studied in recent years [30,31,32]. In this study, terms related to “flavone and flavanol biosynthesis” were enriched in all three comparison groups (S8 cm-vs-S6 cm, S7 cm-vs-S6 cm, and S8 cm-vs-S7 cm) (Figure 7). In addition, “starch and sucrose metabolism”, “anthocyanin biosynthesis”, and “alpha-linolenic acid metabolism” were also differentially enriched pathways. The metabolites involved in those metabolic pathways might be associated with anther dehiscence [3,22,33]. Morphological diagrams of developing stamens also showed pigment accumulation in degrading stomium tissues. Anther SZ cells had nuclear deformation and chromatin condensation and marginalization via PCD. Dong et al. [22] found that overexpression of the plantacyanin gene *OPX* causes indehiscent anthers and that endothecium degeneration in *Arabidopsis* plantacyanin overexpression anthers may be caused by plantacyanin-induced precocious PCD. Similarly, in *Arabidopsis*, the flavonoid transporter FFT is required for anther dehiscence [9]. In the transcriptome database in this study, OXP1 and FFT were upregulated during SZ degeneration (results not shown), and according to the metabolic database, flavanol and anthocyanin accumulation also occurred during SZ degeneration (Figure 8). Furthermore, in this study, ROS-related gene expression indicated that SZ degeneration might be a consequence of ROS accumulation, especially H_2_O_2_ in specific anther cell tissues. Flavonols could prevent ROS from reaching damaging levels and restore impaired fertility [34]. Anthocyanin-deficient mutants had relatively higher ROS levels than the wild type after paraquat treatment, indicating that anthocyanins function as antioxidants to protect against ROS in plants [35]. During the early stage, calcium and diphenyleneiodonium-sensitive reactive oxygen species (ROS) production are required to induce a secondary ROS burst and JA accumulation [36]. Although the cause of accumulation of flavanol and anthocyanin is unclear, ROS-related gene expression suggest that flavanol, anthocyanin, alpha-linolenic acid, and sucrose mediate stomium degeneration by regulating ROS homeostasis in plants.

Plant hormones have significant roles in growth and development and stress resistance. Studies on *Arabidopsis* and crops reveal phytohormones are involved in anther dehiscence, including JAs, auxins, gibberellins, and ethylene [37]. In this study, some auxin signal transduction-related genes (*AUX1*, *IAA*, *GH3*, *ARF*, *SAUR*) were expressed differently in different samples. Auxin regulates early stages of stamen development [38,39]. Auxin localization, biosynthesis, transport, and responses also affect late stages of stamen development, including anther dehiscence, pollen maturation, and preanthesis filament elongation in *Arabidopsis*, in addition to acting on the JAs levels in the initial stages of those processes [16]. In this study, most *AUX1*, *IAA*, *GH3*, *ARF*, and *SAUR* genes were highly expressed in stages S5 cm and S9 cm. This result demonstrated that those genes might regulate the expression of genes involved in SZ lysis during anther development.

JAs contribute to anther dehiscence [40]. Research on the role of JAs in anther dehiscence relies on the JAs signal transduction mutant *coronatine insensitive 1* (*coi1*) [18]. COI1 is an F-box protein that recruits JAs and forms a complex with JAZ proteins (denoted for their ZIM and Jas motifs). The COI1–ligand–JAZ ternary complexes are polyubiquitinated and subsequently degraded by the 26S proteasome, which allows JA signal transduction [18]. In other studies, bHLH factors of JAs pathways antagonize transcription activators, such as MYC2 and the WD-repeat/bHLH/MYB complex, by binding to their target sequence [19,41,42]. In this study, *MYC2* and *JAZs* were downregulated from stage S6 cm to S8 cm (Figure 4). MYC2 and JAZ proteins might repress the transcription of JA-responsive genes as direct regulating factors of SZ degeneration. Defects in anther dehiscence have been observed in mutants of the JA synthesis pathway [15]. JA synthase DAD1 control water transport into the vascular tissues from the anther endothecium, connective tissue, and anther locules, affecting anther dehiscence [43]. In this study, JA synthase was highly expressed in advance, which could provide JA content for the critical stage of anther dehiscence.

Anther dehiscence is also associated with ethylene and gibberellin pathways [44,45]. PhETR2 in petunia regulates SZ degeneration to control anther dehiscence [41], and GA (gibberellin)-dependent anther dehiscence can be cause by defects in secondary thickening or problems of hydration [46]. In this study, several GA and ethylene-related genes, including *DELLA* genes, were highly expressed from stage S6 cm to S8 cm. Gibberellins regulate expression of the JAs biosynthesis gene *DAD1*, and DELLA can act through JAs to control the expression of anther dehiscence-related genes [47]. Thus, the complexity of phytohormones provides a powerful buffer system that is crucial for plants to regulate the complex process of anther dehiscence.

At the cellular level, anther dehiscence likely involves cell wall-degrading enzymes that break down the pectin between cells [48]. Ricinosomes can harbor KDEL-tailed cysteine proteases that are involved in the final stages of cell death [49]. One of the cysteine proteases (SlCysEP) was detected early during tomato anther development in the stomium and epidermal cells surrounding the stomium [50]. In this research, compared with genes of pectin-related enzymes and those associated with anther wall dehydration, those of cysteine proteases had higher expression in the critical stages of anther dehiscence (S6 cm to S8 cm) (Figure 5). Around the SZ of anthers, water transport genes may serve to increase osmotic potential and create a dehydrated environment to provide the final force for anther opening [51]. These processes jointly regulate SZ cell death and anther dehiscence.

Many TFs function as regulatory components of anther dehiscence. Several members of the MYB family, including MYB21, MYB24, MYB26, MYB57, and MYB108, regulate different transcriptional pathways of anther dehiscence. Overexpression those of MYB26 and MYB108 induces anther indehiscence by endothecium secondary thickening, which are also directly regulated by ARF8 and ARF17, respectively [10,12,15]. MYB24 is primarily involved in JA-signal transduction for filament elongation and SZ degradation [52,53]. In addition, MADS-box TF AGAMOUS is also involved in anther development, including anther dehiscence [54,55], and NAC TFs are proposed as regulatory components of endothecium lignification in *Arabidopsis* [56,57]. These results demonstrate that there are several key TFs that directly regulate anther dehiscence, although in different ways. However, the number of TFs identified that regulate anther dehiscence in a given plant remains small. Moreover, relations among TFs have not been fully explored. In this study, transcriptome sequencing data provided a foundation to distinguish regulatory networks and new regulators involved in anther dehiscence (Figure 6). In the transcription data in this study, JAZs and the R2R3 MYB transcription factor MYB21 were also identified. JAZs interact with MYB21 and MYB24, two R2R3-MYB TFs essential for filament elongation, anther dehiscence, and pollen maturation, to mediate JA-regulated male fertility in *Arabidopsis* [19]. This finding is an example of the correlations between different TFs. The weighted expression network based on correlations will provide opportunities to more fully explore the mechanisms of transcription regulation in anther dehiscence.

The fuorochrome-based TUNEL assay is used to detect apoptotic DNA fragmentation [58] and therefore PCD. Lyu et al. [58] found low temperature affected the PCD of tapetum cells and also observed a positive TUNEL signal in stomium cells at low temperatures. In this study, stronger positive TUNEL signals were detected in SZ cells during stages S7 cm and S8 cm, whereas positive signals were not observed in the same tissue during stages S5 cm (Figure 9). The results indicated that SZ cell DNA began to break down at the critical stage of anther dehiscence. Apoptotic cells remained at the edge of the broken region in the later stage. Furthermore, in this study, 11 PCD-related genes had remarkably different expression with JA treatment. CEP1 and CEP2 are KDEL-tailed cysteine endopeptidases, and CEP1 reportedly regulates tapetal PCD [59]. RD21 is also a cysteine proteinase associated with fungal defense [60]. Under heat treatment, expression levels of anther aquaporin genes (*PIPs* and *TIPs*) are significantly correlated with anther dehiscence [61]. However, there are few reports on how the expression of these genes interacts with JA. This study suggested that JA treatment affected anther dehiscence by promoting the expression of genes related to water transport, pectin lysis, and PCD. In summary, on the basis of these analyses, a new understanding of the PCD mechanisms underlying anther SZ degeneration has been obtained.

## 4. Materials and Methods

### 4.1. Plant Materials and Sampling

The Oriental hybrid lily ‘Siberia’ was cultured in a greenhouse at the Lily Resource Preserving Center, Nanjing Agricultural University (Nanjing, China). Day/night temperatures were 26 °C/18 °C, and the relative humidity was 70%. Flowers were divided into nine developmental stages according to the length of flower buds (2 cm to 9 cm corresponded to stages S2 cm to S9 cm). Stomium zone samples were collected by freehand sectioning of anthers and then immediately frozen in liquid nitrogen and stored at −80 °C. All samples were taken from 20 flower buds and pooled to generate one biological replicate. Transcriptome sequencing used samples from stage S5 cm to S9 cm (each stage included three biological replicates), and metabolome sequencing used samples from stage S6 cm to S8 cm (each stage included four biological replicates).

### 4.2. RNA-Seq

Total RNA was extracted using an RNAprep Pure Plant Kit DP441 (Tiangen, Beijing, China), according to the manufacturer’s instructions. RNA integrity was evaluated using an Agilent 2100 Bioanalyzer (Agilent Technologies, Santa Clara, CA, USA). Samples with an RNA integrity number of 7 to 10 were subjected to subsequent analysis. The cDNA libraries were constructed using a HiScript II kit 117 (Vazyme, Nanjing, China). The prepared libraries were sequenced on an Illumina HiSeq TM 2500 platform ((Illumina, San Diego, CA, USA), and 125 bp/150 bp paired-end reads were generated. The database can be found in National Center of Biotechnology Information (NCBI) database under the accession number PRJNA767274.

### 4.3. RNA Data Processing and Analysis

Raw reads of fastqformat were first processed using trimmomatic [62]. To obtain clean reads, original reads and low-quality reads containing poly-n were deleted. After removing the adapters and low-quality sequences, clean reads were assembled into expression sequence tag clusters (contigs) by the Trinity (v2.0.6; http://trinityrnaseq.github.io/, accessed on 2 December 2019), and the transcripts were assembled from scratch. The longest transcript was selected as a single gene for subsequent analysis according to similarity and length. Differentially expressed genes (DEGs) were identified. After annotation, the read counts and FPKM [63] of each unigene were calculated using bowtie2 [64] and express [65], respectively. The threshold for significant differential expression was a *p*-value < 0.05 and fold change >2 or <0.5. Gene Ontology (GO) and Kyoto Encyclopedia of Genes and Genomes (KEGG) pathway enrichment analyses were performed on DEGs using R, based on a hypergeometric distribution [66,67].

### 4.4. Heat Map and Weighted Network Analyses

Tbtools [68] was used to prepare heat maps of the DEGs. Pearson’s correlation coefficients were used to measure the co-expression relations in the Omicshare online program (https://www.omicshare.com/tools/, accessed on 2 June 2021). Only the associations with a *p*-value ≤ 0.05 were selected. In addition, the Omicshare online program (https://www.omicshare.com/tools/Home/Soft/cytoscape2, accessed on 22 June 2021) was used to construct the weighted network.

### 4.5. Sample Preparation and Metabolite Extraction

An accurately weighed sample, 60 mg, was transferred into a 1.5-mL Eppendorf tube. Two small steel balls were added to the tube. The internal standard was 20 μL of 2-chloro-l-phenylalanine (0.3 mg/mL) dissolved in methanol. One milliliter of methanol and water mixture (7:3, *v*/*v*) was added to each sample, and then, the frozen samples were ground at 60 Hz for 2 min and sonicated at ambient temperature for 30 min. Samples were centrifuged at 13,000× *g* rpm at 4 °C for 10 min. The supernatant, 300 mL, was removed and freeze-dried. A mixture of methanol and water (1:4, *v*/*v*), 400 mL, was added to each dry sample, and samples were then rotated and exposed to ultrasonic treatment. Samples were centrifuged at 13,000× *g* rpm at 4 °C for 5 min. Samples, 150 μL, were collected from each tube using a crystal syringe, passed through a 0.22 μm filter, and transferred to LC (liquid chromatograph) vials. Vials were stored at −80 °C until LC–MS (liquid chromatograph–mass spectrometer) analysis.

### 4.6. Liquid Chromatography−Tandem Mass Spectrometry

Metabolic profiles in both the ESI-positive and ESI-negative ion modes were analyzed by using an Acquity UHPLC system (Waters Corporation, Milford, MA, USA) and an AB Sciex Triple TOF 5600 System (AB Sciex, Framingham, MA, USA). An Acquity UPLC BEH C18 column (1.7 μm, 2.1 × 100 mm) was used in both positive and negative modes. The binary gradient elution system consisted of water (A, containing 0.1% formic acid, *v*/*v*) and acetonitrile (B, containing 0.1% formic acid, *v*/*v*).

Compounds were separated using the following gradient: 0 min, 5% B; 2 min, 20% B; 4 min, 25% B; 9 min, 60% B; 14 min, 100% B; 18 min, 100% B; 18.1 min, 5% B, and 19.5 min, 5% B. The flow rate was 0.4 mL/min, and the column temperature was 45 °C. During the analysis, all samples were kept at 4 °C.

Mass range was from 66.7 to 1000.5 *m*/*z*. Resolution of the full MS scan was set to 70,000, and resolution of the HCD MS/MS scan was set to 35,000. Collision energy I was set to 10, 20, and 40 ev. Operation of the mass spectrometer was as follows: spray voltage, 3000 V (+) and 2500 V (−); sheath gas velocity, 45 arbitrary units; auxiliary gas flow rate, 15 arbitrary units; and capillary temperature, 350 °C.

### 4.7. Data Preprocessing and Statistical Analyses

Metabolites were identified using the data processing software of progenesis QI 2.0 (Waters Corporation, Milford, MA, USA) based on public databases such as http://www.hmdb.ca/ and http://www.lipidmaps.org/ (accessed on 23 March 2020) and also self-built databases.

Differential metabolites were selected according to the combination of statistically significant variable influence projection threshold (VIP) values obtained from an OPLS-DA (orthogonal partial least squares–discrimination analysis) [69] model and *p*-values obtained from a two-tailed Student’s *t*-test of standardized peak area. Metabolites with VIP values >1.0 and *p*-values <0.05 were regarded as differential metabolites.

### 4.8. TUNEL Assay

Paraffin wax-embedded samples were sectioned to 8-µm thickness using a Leica RM2235 microtome (Leica, Wetzlar, Germany). Nick-end labeling of the fragmented DNA was performed using a One Step TUNEL Apoptosis Assay Kit (Beyotime, Beijing, China) according to the manufacturer’s protocols. Samples were dewaxed in 100% xylene and dehydrated in a graded ethanol wash (100%, 95%, 85%, 70%, 50%, 0%), with 3 min in each step. Samples were incubated in 20 µg/mL Proteinase K solution for 15 min and then washed in PBS (Phosphate Buffered Saline) twice. TUNEL reaction mixture, 50 µL, was added to the sample cells, which were then incubated for 1 h at 37 °C in the dark. Samples treated without reaction mixture were the controls. Images were taken using a laser scanning confocal microscope (LSM800, Zeiss, Oberkochen, Germany).

### 4.9. Reverse-Transcription Quantitative PCR

Total RNA isolated from the SZ of stage S6 cm to S9 cm treated with 0 or 50 μM MeJA (Bioduly, Nanjing, China) for 48 h. The cDNA was prepared using a HiScript II kit 117 (Vazyme, Nanjing, China). Quantitative RT-qPCR reactions were conducted using ChamQ Universal SYBR qPCR Master Mix (Vazyme, Nanjing, China) following the manufacturer’s protocol. Primers for the genes were designed using Primer express 3.0 software (Table S6). Gene expression levels were normalized using the reference gene 18S [5], and values were calculated using the 2^−ΔΔCt^ method [70,71]. Expression values were obtained from three biological experiments. SPSS 17.0 statistical software was used to determine statistical significance.

## 5. Conclusions

**Figure 11:**
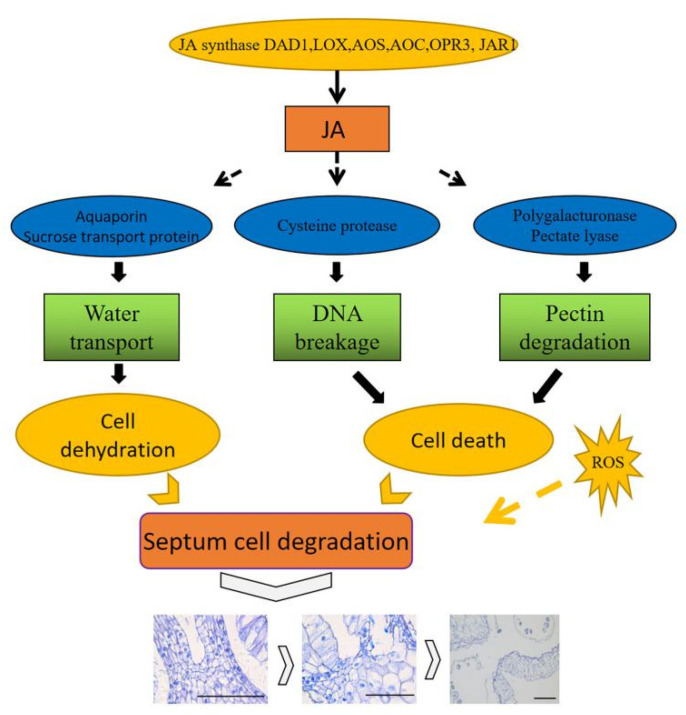
Model of anther dehiscence. JA: Jasmonates; DAD1: lipase defective of anther dehiscence; LOX: lipoxygenase; AOS: allene oxide synthase; AOC: allene oxide cyclase; OPR3: OPDA reductase. JAR1: jasmonic acid synthetase; ROS: reactive oxygen species. Jasmonate synthase regulates the level of JA. Jasmonate downregulates the expression of genes associated with water transport, PCD, and pectin degradation, to induce cell dehydration and death. In addition, ROS are also involved in SZ degradation. Arrows indicate induction, and the dashed line indicates putative induction. Bar = 200 μm.

In summary, SZ degeneration is essential for anther dehiscence. On the basis of the transcriptomic and metabolomic analysis of SZ degeneration, signaling of phytohormones, including JAs, affected the transcript abundances of the genes associated with pectinase, water and sucrose transport, and cysteine proteinase, which led to stomium cell death by regulating water transport, DNA breakage, and pectin degradation (Figure 11). Thus, a morphological process of anther SZ degeneration in lily is proposed. This work also provides data to help reveal the regulatory mechanisms of SZ degeneration in plants to improve crop breeding.

## Supplementary Materials

The following are available online at https://www.mdpi.com/article/10.3390/ijms222212124/s1.
