# Supplementary figures and images for: Transcriptome and Metabolome Analyses Provide Insights into the Stomium Degeneration Mechanism in Lily
Source: Int J Mol Sci. 2021 Nov 9;22(22):12124. doi: 10.3390/ijms222212124 (PMC8619306; doi:10.3390/ijms222212124)

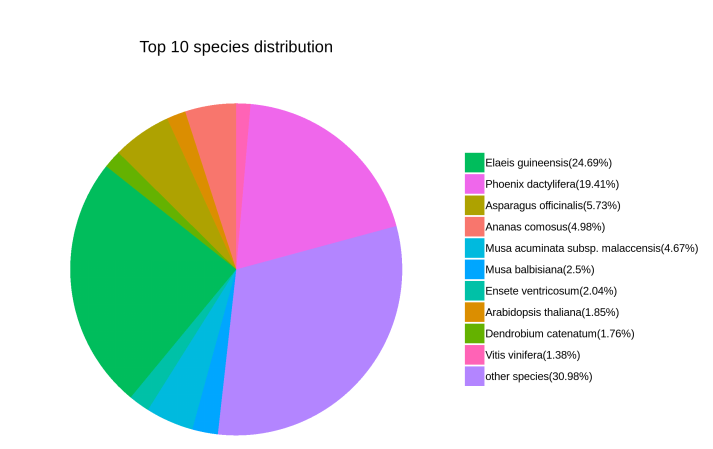

Supplement: Supplementary file 1 [file ijms-22-12124-s001.zip › Figure.S1 The disstribution of annotated species.jpg]

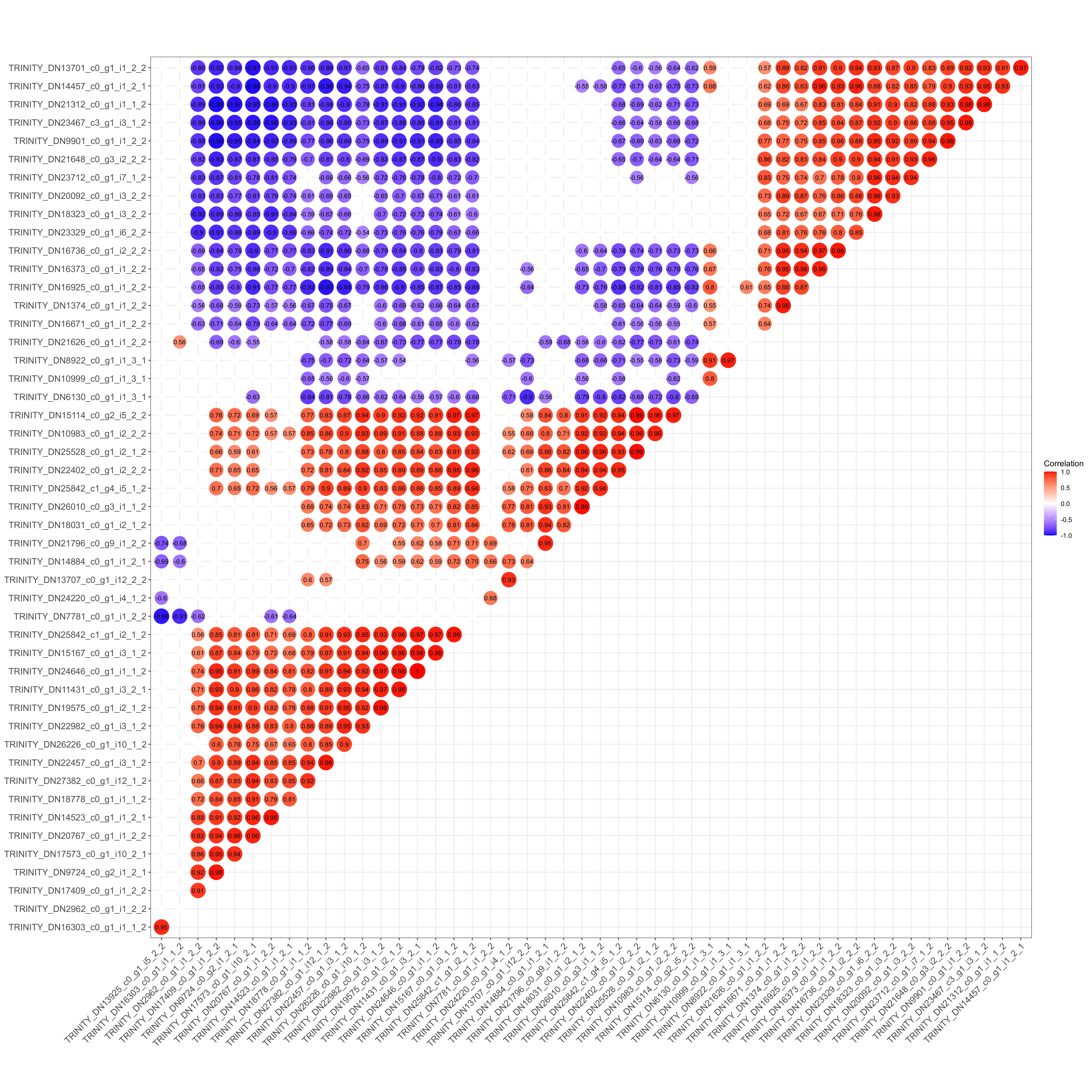

Supplement: Supplementary file 1 [file ijms-22-12124-s001.zip › Figure.S2 Correlation analysis of the 49 TFs.jpg]
